# The Influence of AA29504 on GABA_A_ Receptor Ligand Binding Properties and Its Implications on Subtype Selectivity

**DOI:** 10.1007/s11064-021-03475-y

**Published:** 2021-11-02

**Authors:** Sylvia Sikstus, Ali Y. Benkherouf, Sanna L. Soini, Mikko Uusi-Oukari

**Affiliations:** Integrative Physiology and Pharmacology, Institute of Biomedicine, University of Turku, Kiinamyllynkatu 10, 20014 Turku, Finland

**Keywords:** GABA, Muscimol, AA29504, GABA_A_ receptor, EBOB, Allosteric modulation

## Abstract

The unique pharmacological properties of δ-containing γ-aminobutyric acid type A receptors (δ-GABA_A_Rs) make them an attractive target for selective and persistent modulation of neuronal excitability. However, the availability of selective modulators targeting δ-GABA_A_Rs remains limited. AA29504 ([2-amino-4-(2,4,6-trimethylbenzylamino)-phenyl]-carbamic acid ethyl ester), an analog of K^+^ channel opener retigabine, acts as an agonist and a positive allosteric modulator (Ago-PAM) of δ-GABA_A_Rs. Based on electrophysiological studies using recombinant receptors, AA29504 was found to be a more potent and effective agonist in δ-GABA_A_Rs than in γ2-GABA_A_Rs. In comparison, AA29504 positively modulated the activity of recombinant δ-GABA_A_Rs more effectively than γ2-GABA_A_Rs, with no significant differences in potency. The impact of AA29504's efficacy- and potency-associated GABA_A_R subtype selectivity on radioligand binding properties remain unexplored. Using [^3^H]4'-ethynyl-4-n-propylbicycloorthobenzoate ([^3^H]EBOB) binding assay, we found no difference in the modulatory potency of AA29504 on GABA- and THIP (4,5,6,7-tetrahydroisoxazolo[5,4-c]pyridin-3-ol)-induced responses between native forebrain GABA_A_Rs of wild type and δ knock-out mice. In recombinant receptors expressed in HEK293 cells, AA29504 showed higher efficacy on δ- than γ_2_-GABA_A_Rs in the GABA-independent displacement of [^3^H]EBOB binding. Interestingly, AA29504 showed a concentration-dependent stimulation of [^3^H]muscimol binding to γ_2_-GABA_A_Rs, which was absent in δ-GABA_A_Rs. This was explained by AA29504 shifting the low-affinity γ_2_-GABA_A_R towards a higher affinity desensitized state, thereby rising new sites capable of binding GABA_A_R agonists with low nanomolar affinity. Hence, the potential of AA29504 to act as a desensitization-modifying allosteric modulator of γ2-GABA_A_Rs deserves further investigation for its promising influence on shaping efficacy, duration and plasticity of GABA_A_R synaptic responses.

## Introduction

γ-Aminobutyric acid type A receptors (GABA_A_R), members of the Cys-loop ligand-gated ion channels superfamily, are the major sites for fast-acting synaptic inhibition in the mammalian brain [1, 2]. These heteropentameric protein complexes contain an inherent chloride channel that opens upon the binding of GABA, where chloride ion influx hyperpolarizes the membrane potential, thereby inhibiting the neuron [1, 3]. Numerous clinically significant drugs, including benzodiazepines, barbiturates, neurosteroids, and anesthetics, have been shown to positively modulate GABA_A_R function [4]. GABA_A_R protein subunits are encoded by 19 distinct genes: α1‐α6, β1‐β3, γ1‐γ3, δ, ε, π, θ, and ρ1‐ρ3 [1]. Each GABA_A_R subunit is comprised of a large extracellular N terminus, four transmembrane domains (TM1–4), one extracellular TM2–3 loop, two intracellular loops (TM1–2 and TM3–4), and an extracellular C terminus [5]. The majority of receptor subtypes are composed of α, β and γ subunits with a stoichiometry of 2α:2β:1γ [6, 7]. These subtypes are mostly sensitive to benzodiazepines and reside in post‐synaptic sites where they mediate fast synaptic phasic inhibition [1]. Receptor combinations in which γ2 is substituted with δ (2α:2β:1δ) are found in extra‐ and perisynaptic membranes. δ-containing GABA_A_Rs (δ‐GABA_A_Rs) are benzodiazepine-insensitive and display a high affinity for GABA, which enables them to be activated by low GABA concentrations to mediate a slow-desensitizing tonic inhibition [8–11]. Due to the unique functional and pharmacological properties of δ‐GABA_A_Rs, they represent an attractive drug target for selective and persistent modulation of neuronal excitability. The therapeutic potential of δ‐GABA_A_Rs has been studied in several disorders such as epilepsy [12, 13], schizophrenia [14], stroke [15], tremors [16], stress [17, 18] and alcohol withdrawal [19, 20]. However, in comparison to γ2-GABA_A_Rs, the availability of selective allosteric modulators targeting δ-GABA_A_Rs remains limited.

**Fig. 1:**
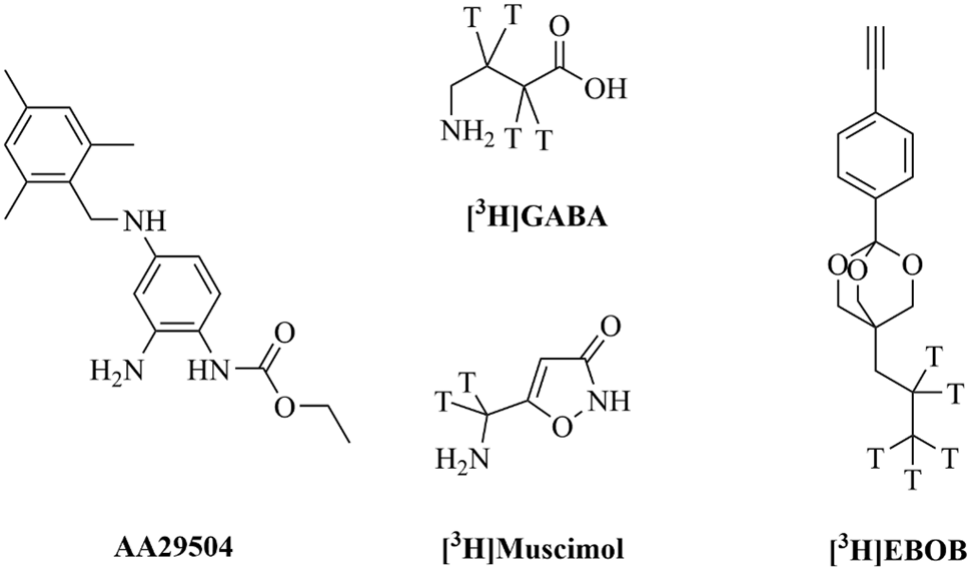
Chemical structures of AA29504 and the radioligands used in this study. T indicates the tritium (^3^H) radiolabel position

DS2 (4-chloro-N-[2-(2-thienyl)imidazo[1,2-a]pyridin-3-yl]benzamide), a selective positive allosteric modulator of α4/6βδ receptors, is a widely used pharmacological tool to probe δ-GABA_A_R-mediated responses [21, 22]. It has been demonstrated to improve stroke recovery in vivo*,* but it showed limited brain bioavailability [23]. AA29504 ([2-amino-4-(2,4,6-trimethylbenzylamino)-phenyl]-carbamic acid ethyl ester) (Fig. 1), a structural analog of the voltage-gated potassium channel (KCNQ) opener retigabine, acts as an allosteric agonist and a positive allosteric modulator (Ago-PAM) of δ-GABA_A_Rs at low micromolar concentrations [24–27]. In relation to DS2, AA29504 exhibits a superior brain permeability (60 min post 2 mg/kg, s.c.in mice resulted in 1 μM brain concentration) and has been shown to alleviate anxiety, stress and cognitive deficits in phencyclidine (PCP) rat model of schizophrenia [24, 25, 28]. These behavioral effects were associated with AA29504 modulation of extrasynaptic GABA_A_Rs in the same studies, making it a promising tool to explore the role of extrasynaptic GABA_A_R transmission in the CNS. The functional properties of AA29504 have been examined earlier using several electrophysiological techniques, including acutely prepared slices, cultured neurons and recombinant *Xenopus* oocyte/ stable HEK293 Flp-In™ systems for various GABA_A_R subtypes [24–27]. AA29504 at 1 μM concentration was found to enhance both phasic and tonic currents induced by GABA_A_R superagonist THIP (4,5,6,7-tetrahydroisoxazolo[5,4-c]pyridin-3-ol) in rat cortical brain slices and mice dentate gyrus granule cells [24, 25]. In recombinant receptors, AA29504 was a more potent and effective agonist in δ-GABA_A_Rs than in γ2-GABA_A_Rs [27]. On the other hand, AA29504 positively modulated the activity of recombinant δ-GABA_A_Rs more effectively than that of γ2-GABA_A_Rs, but no significant differences were noted in terms of potency [24, 27]. However, ligand-receptor interactions governing the complex AA29504's efficacy- and potency-associated subtype selectivity remain unexplored. Radioligand binding assay is a valuable technique to elucidate this and to quantify the molecular parameters derived from single or multiple ligand-bound states [29, 30]. Earlier binding studies on native GABA_A_Rs expressed in rat brain membranes reported that the analogue retigabine increased the binding affinity to GABA and vice versa [31]. Hence, we hypothesize AA29504's potential to selectively modulate the binding of specific receptor populations by shifting their binding affinity or by other mechanisms, which deserve experimental investigation. This would contribute to the pharmacological characterization of AA29504's selective modulatory activity and its interactions with GABA_A_Rs agonists, as well as the interpretation of its GABA_A_R-mediated effects in vivo.

In this study, we implemented radioligand binding assays to examine the allosteric modulatory behavior of AA29504 and its influence on agonist binding properties in native and recombinant GABA_A_Rs. The selectivity of AA29504 to δ-GABA_A_Rs was confirmed using wild-type (WT) and δ subunit knockout (δKO) C57BL/6 J mice forebrains, as well as recombinant receptors expressed in human embryonic kidney 293 (HEK293) cell line. Radioligands employed (Fig. 1) were [^3^H]4'-ethynyl-4-n-propylbicycloorthobenzoate ([^3^H]EBOB), a non-competitive blocker of GABA-gated chloride channel [32, 33], the neurotransmitter [^3^H]GABA, and [^3^H]muscimol, a universal GABA_A_R agonist with exceptionally high affinity to δ-GABA_A_Rs [34–36].

## Materials and Methods

### Animals

Wild‐type (C57BL/6J, WT; RRID: IMSR JAX:000,664), and GABA_A_R δ subunit knockout (C57BL/6J, δKO; RRID: MGI:3,639,693) mice (age: 3–12 months, both sexes; weight: 19–32 g) were a kind gift from Dr. Martin Wallner (UCLA: University of California, Los Angeles, Los Angeles, CA). The δKO mice were originally produced and validated at Harmonics Lab by injecting ES cells into C57BL/6J blastocysts and backcrossing them with C57BL/6J mice for at least ten generations (Jackson Laboratories, stock No. 000664) [12]. The mice were maintained on a hybrid C57BL/6–129 Sv background and genotyped by Southern blot analysis as previously reported [12]. Briefly, BamHI-digested mouse tail DNA samples were hybridized with 830-bp PCR product (probe D). Southern blot of BamHI digested DNA indicated that probe D hybridized to a 6.6-kb BamHI fragment from the wild-type δ gene and a 7.7-kb BamHI fragment from the targeted allele. All animals were housed in standard conditions (12:12 h light: dark cycle at 21 ± 1 °C and humidity 65%) with access to Rodent Lab Chow #5001 and filtered tap water ad libitum. Mice were euthanized by decapitation, their fore/midbrains were dissected (loosely referred as forebrain), frozen on dry ice, and stored at -70 °C. All experimental procedures in this study complied with protocols approved by the Animal Experiment Board in Finland and UCLA Chancellor’s Animal Research Committee (Animal Welfare Approval Number: A3196‐01).

### Reagents

[^3^H]Muscimol (22 Ci/mmol) and [^3^H]EBOB (48 Ci/mmol) were purchased from PerkinElmer Life and Analytical Sciences (Boston, MA, USA). [^3^H]GABA (30 Ci/mmol) was purchased from Moravek Biochemicals (Brea, CA, USA). Unlabeled GABA and picrotoxin were from Sigma Chemicals Co. (St. Louis, MO, USA). AA29504 and THIP were from Tocris Biosciences (Bristol, UK).

### Preparation of Brain Membranes

WT and δKO forebrain membranes were prepared using the method of Squires and Saederup [37] as modified by Uusi-Oukari et al. [38]. Homogenized and washed membranes were suspended in 10 mM Tris–HCl, pH 7.4 and stored at -70 °C. Prior to binding experiments, the frozen membrane suspensions were thawed, centrifuged at 20,000 g for 10 min at + 4 °C, and resuspended in assay buffer.

### Recombinant GABA_A_ Receptor Expression in HEK293 cells

Human embryonic kidney (HEK) 293 cells were maintained at + 37 °C/10% CO_2_ in Dulbecco’s Modified Eagle’s Medium (DMEM) (Gibco, Gaithersburg, MD, USA), supplemented with 3.7 g/L NaHCO_3_, 10% Fetal Bovine Serum (FBS) (Gibco, Gaithersburg, MD, USA), 50,000 U/L penicillin and 50 mg/L streptomycin (Sigma-Aldrich, St Louis, MO, USA). The cells were divided and plated on 10 cm culture dishes for binding assays 24 h before transfection. HEK293 cells were transfected with rat cDNAs (α1, α6, β2, β3, γ2S, δ) in pRK5 plasmids [39] under the control of cytomegalovirus (CMV) promoter using calcium phosphate transfection method essentially as described by Lüddens and Korpi [40]. The plasmids were used in 1:1 and 1:1:1 ratios for transfections containing 2 [(α6) + (β3)] or 3 [(α1 or α6) + (β2 or β3) + (γ2S or δ)] different subunits, respectively (5 μg of each plasmid DNA for a 10 cm plate). The cells were incubated at 37 °C/10% CO_2_ for 24 h post-transfection. Old culture medium was replaced with fresh medium and the incubation resumed for another 24 h. The cells were harvested 48 h post-transfection. Culture medium was removed and the cells were detached from the plates by pipetting in ice-cold buffer containing 50 mM Tris-citrate or 10 mM Tris–HCl, 0.15 M NaCl, 2 mM EDTA, pH 7.4 and centrifuged at 20,000×*g* for 10 min at + 4 °C. The resulting pellets were finally suspended in assay buffer and used directly in binding assays.

### Measurement of [^3^H]muscimol and [^3^H]GABA Binding

The binding of [^3^H]muscimol (2 nM) and [^3^H]GABA (5 nM) were measured in assay buffer (10 mM Tris–HCl, pH 7.4) at room temperature (22 ºC) in a total volume of 300 µl. Individually pooled triplicate membrane samples (3 total binding and 3 non‐specific binding) were incubated with shaking for 15 min. The effect of AA29504 on binding was determined in the presence of various concentrations of AA29504. Non-specific binding was determined in the presence of 100 µM GABA. The incubation was terminated by filtration of the samples with a Brandel Cell Harvester (model M-24, Gaithersburg, MD, USA) onto Whatman GF/B filters (Whatman International Ltd., Maidstone, UK).

The samples were rinsed twice with 4–5 ml of ice-cold assay buffer. Filtration and rinsing steps took a total time of 15 s. The filters were air-dried and immersed in 3 ml of Optiphase HiSafe 3 scintillation fluid (Wallac, Turku, Finland) and vortexed. The radioactivity was determined in a Wallac model 1410 liquid scintillation spectrometer (Wallac, Turku, Finland). The average specific counts per minute (CPM) and % specific [^3^H]muscimol (2 nM) binding to the membrane homogenates were as follows: WT (1649 CPM, 88%), δKO (1566 CPM, 84%), α1β2γ2 (366 CPM, 58%), α6β2γ2 (256 CPM, 54%) and α6β2δ (191 CPM, 47%). For [^3^H]GABA (5 nM) the binding values were: WT (516 CPM, 68%) and δKO (292 CPM, 55%).

The effect of AA29504 on the association and dissociation of [^3^H]muscimol binding was measured essentially as described by Benkherouf et al. [41]. Saturation analysis of [^3^H]muscimol was performed essentially as described by Uusi-Oukari and Korpi [42]. Triplicate samples of the membranes were incubated in assay buffer with a concentration series of [^3^H]muscimol (1–50 nM) at room temperature (22 ºC) for 60 min in the absence and presence of 10 µM AA29504. Non-specific binding was determined in the presence of 100 µM GABA. The incubation was terminated by filtration and the radioactivity of the air-dried filters was measured using a scintillation spectrometer as described above.

### Measurement of [^3^H]EBOB Binding

The displacement of 1 nM [^3^H]EBOB binding was measured in [^3^H]EBOB assay buffer (50 mM Tris–HCl, pH 7.4, 120 mM NaCl) at room temperature (22 ºC) in a total volume of 400 µl in the absence and presence of various concentrations of GABA or THIP with and without 10 µM AA29504. Triplicate samples were incubated with shaking for 2 h. Non-specific binding was determined in the presence of 100 µM picrotoxin. The incubations were terminated as described above for [^3^H]muscimol binding. The average CPM and % specific [^3^H]EBOB (1 nM) binding to the membrane homogenates were as follows:WT (889 CPM, 77%), δKO (1020 CPM, 79%), α6β3γ2 (1055 CPM, 89%), α6β3δ (402 CPM, 66%), α6β3: (1200 CPM, 90%).

### Protein Measurement

In all radioligand binding experiments, the average protein concentrations were 0.8 mg/ml for WT and 0.9 mg/ml for δKO forebrain membranes. These were determined with Bio-Rad Coomassie blue dye-based protein assay kit (Hercules, CA, USA) as per the manufacturer’s protocol.

### Data Analysis

GraphPad Prism software (GraphPad, San Diego, CA, USA) was used for nonlinear least squares curve-fitting and statistical testing of association, dissociation, saturation binding and radioligand displacement data. The association and dissociation curves were used for the estimation of association (K_on_) and dissociation (K_off_) rate constants. Saturation binding curves were used for the estimation of total number of high-affinity binding sites (B_max_), and equilibrium dissociation constants (K_D_). Radioligand displacement values were fitted to a sigmoidal dose–response (variable Hill Slope) curve for the estimation of the half-maximal inhibitory concentration (IC_50_):

$${\text{Y}} = {\text{Bottom}} + ({\text{Top - Bottom}})/(1 + ({\text{IC}}50/{\text{X}})^{{{\text{HillSlope}}}} )$$

where Y is the percentage of control binding, Bottom = 0 when non-specific binding is subtracted from all binding values, Top is the maximum radioligand binding in the absence of test compound, and X is the test compound concentration. Statistical comparisons were made with One-way ANOVA, Two-way ANOVA or Brown-Forsythe and Welch ANOVA followed by the relevant (Tukey’s or Dunnett’s) post hoc tests for multiple comparisons. All data were expressed as means ± SEM and p-values of less than 0.05 were considered significant. The study samples were not randomized and analysis was performed in a parallel unblinded mode.

## Results

### AA29504 Modulation of GABA- and THIP-Induced [^3^H]EBOB Displacement

**Fig. 2:**
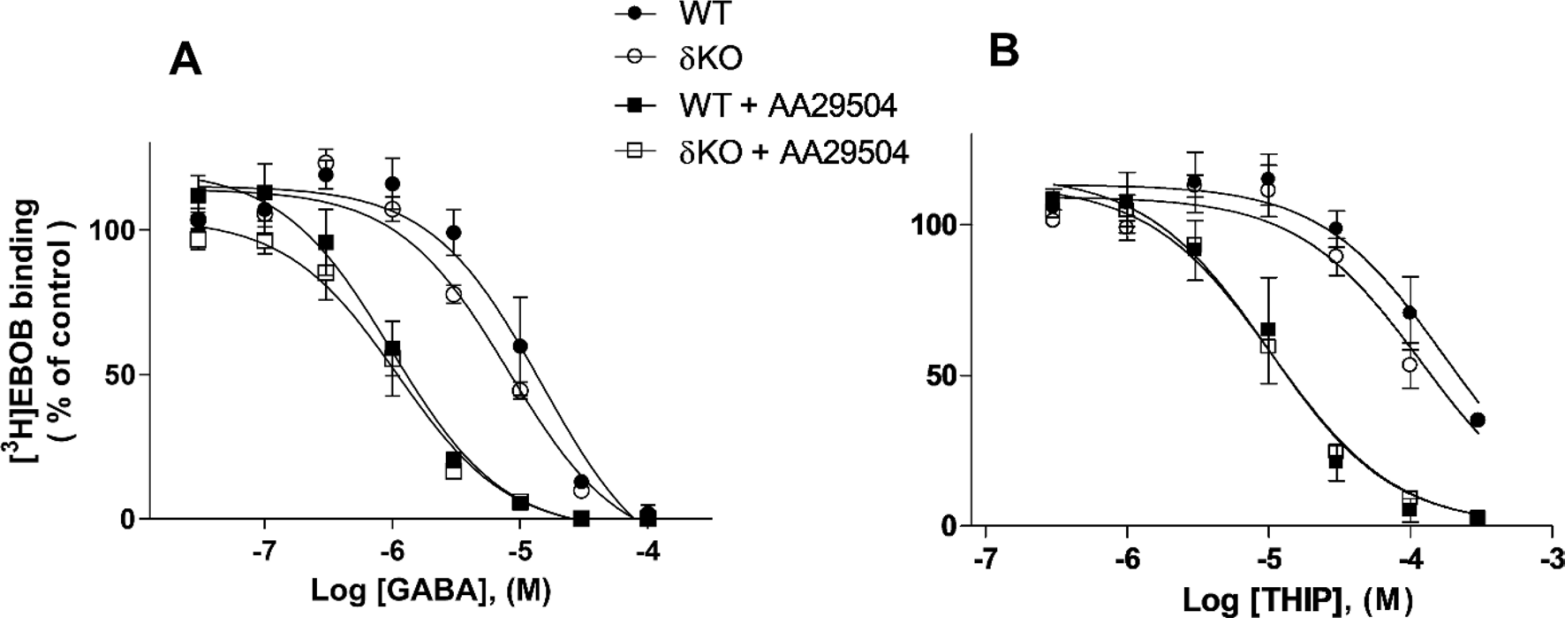
AA29504 positive modulation of GABA- and THIP- induced 1 nM [^3^H]EBOB displacement in WT and δKO mice forebrain membranes. Displacement curves of [^3^H]EBOB binding as % of control with 7–8 concentrations of GABA (**A**) and THIP (**B**) in the absence or presence of 10 µM AA29504. The control is basal [^3^H]EBOB binding in the absence of GABA, THIP and AA29504. The radioligand displacement by AA29504 was significant in WT (p < 0.05) and δKO mice (p < 0.05), with no potency difference between the mouse lines (p > 0.05). All values represent the mean ± SEM, n = 3 independent experiments using triplicate membrane samples pooled individually from each mouse line’s forebrain

[^3^H]EBOB binding assay was initially carried out to evaluate AA29504's allosteric modulatory activity on native GABA_A_Rs expressed in WT and δKO forebrain membranes. A concentration series of GABA- and THIP- induced [^3^H]EBOB displacement was performed in the absence or presence of AA29504. As highlighted in Fig. 2, the inclusion of 10 µM AA29504 produced a leftward shift of [^3^H]EBOB displacement curves in both mouse lines. AA29504 decreased the IC_50_ of GABA-induced [^3^H]EBOB displacement from 15.9 ± 5.0 μM to 1.0 ± 0.2 μM in WT mice (p < 0.05) and from 8.4 ± 1.8 µM to 1.2 ± 0.5 µM in δKO mice (p < 0.05). Furthermore, AA29504 decreased the IC_50_ of THIP-induced [^3^H]EBOB displacement from 0.20 ± 0.11 mM to 15.8 ± 6.5 µM in WT mice (p < 0.01) and from 0.22 ± 0.05 mM to 12.1 ± 1.5 µM in δKO mice (p < 0.05, One-way ANOVA followed by Tukey’s post hoc test). There were no significant differences between WT and δKO mouse binding values in the effects of GABA or THIP on forebrain GABA_A_Rs in the absence or presence of AA29504 (p > 0.05, Two-way ANOVA followed by Tukey’s post hoc test).

### The Direct Actions of AA29504 on [^3^H]EBOB Binding to Recombinant GABA_A_ Receptors

**Fig. 3:**
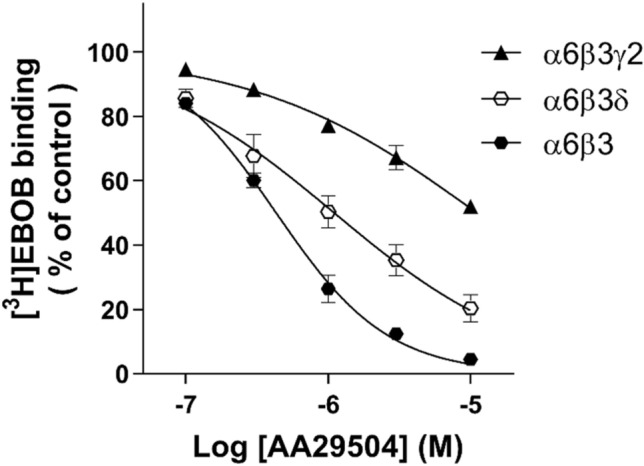
GABA-independent displacement curves of 1 nM [^3^H]EBOB binding to recombinant α6β3, α6β3γ2 and α6β3δ GABA_A_Rs expressed in HEK293 cells with 5 concentrations of AA29504. The control is basal [^3^H]EBOB binding in the absence of AA29504. AA29504 displacement potency followed the rank order: α6β3 > α6β3δ > α6β3γ2 (p < 0.05). All values represent means ± SEM, n = 3–6 independent transfections and experiments performed in triplicate

We assessed the potential of AA29504 to directly displace [^3^H]EBOB binding to recombinant GABA_A_Rs expressed in HEK293 cells as an indicator for allosteric agonist activity. The receptor subunit combinations α6β3γ2, α6β3δ, and α6β3 were selected to further examine the influence of γ and δ subunits on [^3^H]EBOB binding displacement by AA29504. The results indicate that AA29504 was able to displace [^3^H]EBOB binding to all three receptor subtypes in a concentration-dependent manner (Fig. 3). This GABA-independent radioligand displacement was evident already at a nanomolar range for α6β3 (≥ 100 nM), α6β3δ (≥ 100 nM) and α6β3γ2 (≥ 300 nM) receptors where the calculated IC_50_ values for AA29504 were 0.4 ± 0.02 μM, 1.1 ± 0.2 μM and 11.1 ± 1.4 μM, respectively. Statistical analysis revealed a significant difference with regard to AA29504 potency on the tested recombinant receptors as it followed the rank order: α6β3 > α6β3δ > α6β3γ2 (p < 0.05, Brown-Forsythe and Welch ANOVA followed by Dunnett’s post hoc test).

### AA29504 Stimulation of [^3^H]muscimol and [^3^H]GABA Binding to Native GABA_A_ Receptors

**Fig. 4:**
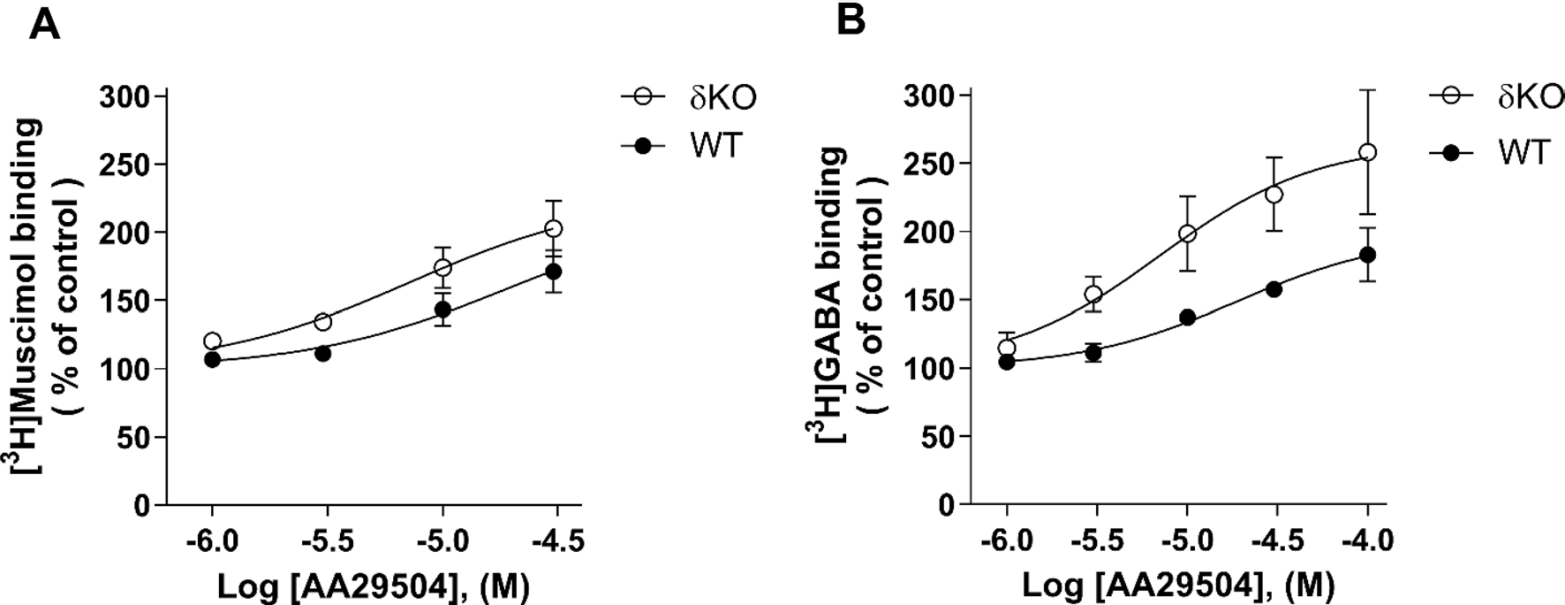
The effects of AA29504 on 2 nM [^3^H]muscimol (**A**) and 5 nM [^3^H]GABA (**B**) binding to WT and δKO mice forebrain membranes. AA29504 was significantly more potent in stimulating [^3^H]muscimol (p < 0.01) and [^3^H]GABA (p < 0.001) binding in δKO than in WT mice. The values represent [^3^H]muscimol binding as % of control with 4 concentrations of AA29504, where the control is basal [^3^H]muscimol **(A)** or [^3^H]GABA **(B)** binding in the absence of AA29504 (mean ± SEM, n = 3 independent experiments using triplicate membrane samples pooled individually from each mouse line’s forebrain)

We further examined the modulatory effects of AA29504 on the high-affinity agonist binding to native GABA_A_Rs expressed in WT and δKO mice. The binding of [^3^H]muscimol and [^3^H]GABA was measured at room temperature (22 °C) with increasing concentrations of AA29504, where individually pooled forebrain membrane samples were incubated for 15 min. Figure 4 shows that AA29504 produced a concentration-dependent stimulation in [^3^H]muscimol and [^3^H]GABA binding to both WT and δKO mice forebrains. Two-way ANOVA followed by Tukey's post hoc analysis indicated that AA29504 was more potent in stimulating [^3^H]muscimol (p < 0.01) and [^3^H]GABA (p < 0.001) binding in δKO than in WT mice.

### AA29504 Stimulation of [^3^H]muscimol Binding to Recombinant GABA_A_ Receptors

**Fig. 5:**
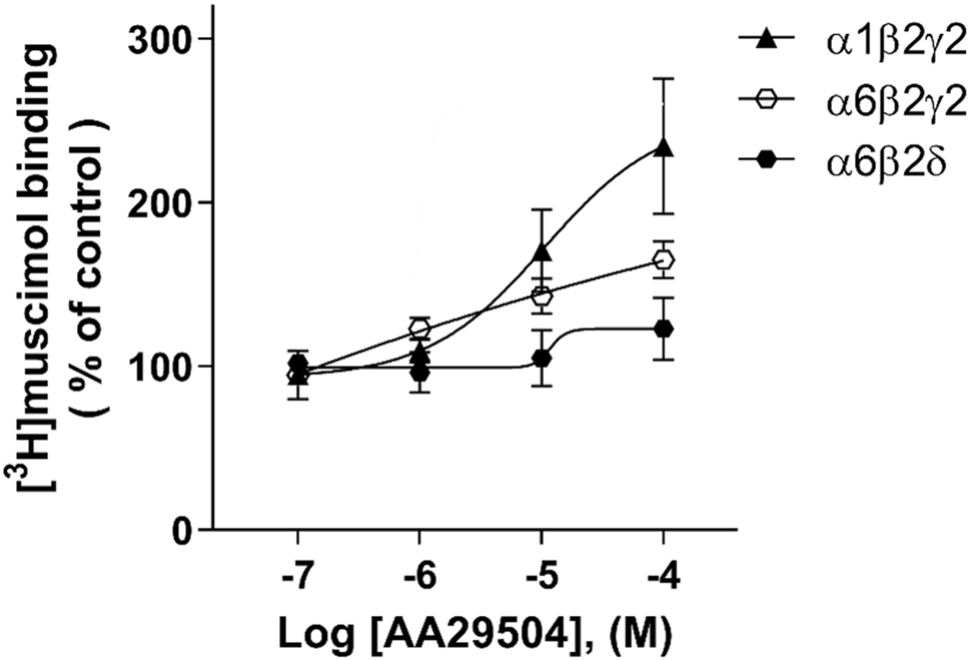
The effect of AA29504 on 2 nM [^3^H]muscimol binding to recombinant α1β2γ2, α6β2γ2 and α6β2δ GABA_A_Rs expressed in HEK293 cells. AA29504 potentiation of [^3^H]muscimol binding was significant in α1β2γ2 and α6β2γ2 (p < 0.05), while absent in α6β2δ recombinant GABA_A_Rs (p > 0.05)**.** The values represent [^3^H]muscimol binding as % of control with 4 concentrations of AA29504, where the control is basal [^3^H]muscimol binding in the absence of AA29504 (mean ± SEM, n = 3–4 independent transfections and experiments performed in triplicate)

Under the same conditions performed for native GABA_A_Rs expressed in WT and δKO mice forebrains, we compared the role of γ and δ subunits on AA29504 modulation of [^3^H]muscimol binding in recombinant α1β2γ2, α6β2γ2, and α6β2δ receptors expressed in HEK293 cells. As illustrated in Fig. 5, AA29504 stimulated [^3^H]muscimol binding to α1β2γ2 and α6β2γ2 receptor subtypes in a concentration-dependent manner (p < 0.05). In contrast, it had no significant effect on the binding to α6β2δ receptor subtype (Two-way ANOVA followed by Tukey’s post hoc test).

### The Effect of AA29504 on [^3^H]muscimol Binding Kinetics

**Fig. 6:**
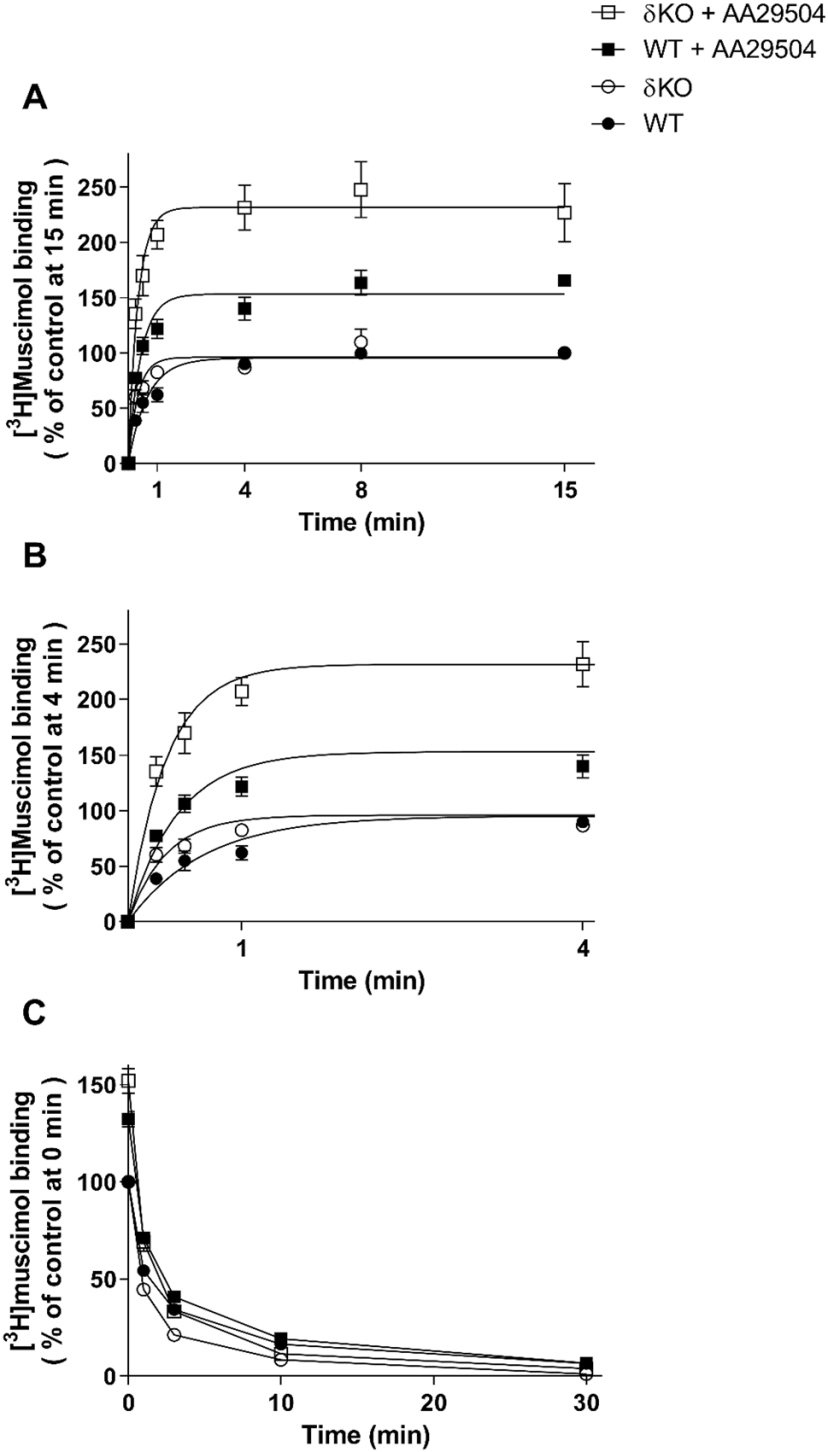
The association (**A**) and dissociation (**C**) of 2 nM [^3^H]muscimol with WT and δKO mice forebrain membranes in the absence or presence of 10 µM AA29504**.** Insert of Fig. 6A association analysis where 0–4 min time range is highlighted (**B**). The co-incubation with AA29504 did not produce significant changes in the association (K_on_) and dissociation (K_off_) rate constants in either mouse line (p > 0.05). The values are expressed as % of control binding at 15 min for association and 0 min for dissociation, where the control is the maximal [^3^H]muscimol basal binding in the absence of AA29504 (mean ± SEM, n = 3 independent experiments using triplicate membrane samples pooled individually from each mouse line’s forebrain)

We questioned whether AA29504 stimulation of [^3^H]muscimol binding is due to alterations in receptor-ligand binding kinetics as we assessed the influence of AA29504 on [^3^H]muscimol association and dissociation rates in WT and δKO forebrain membranes. The results indicate that [^3^H]muscimol association at 22 °C was faster in δKO compared to WT in the absence of AA29504, where the calculated association rate constants K_on_ were 5.9 ± 0.6 × 10^8^ M^−1^ × min^−1^ and 2.2 ± 0.3 × 10^8^ M^−1^ × min^−1^, respectively (mean ± SEM, n = 3) (p < 0.05, One-way ANOVA followed by Tukey's post hoc test). Co-incubation with AA29504 did not significantly affect [^3^H]muscimol K_on_ in either δKO (4.8 ± 0.9 × 10^8^ M^−1^ × min^−1^) or WT mice (3.9 ± 1.1 × 10^8^ M^−1^ × min^−1^) (mean ± SEM, n = 3), but it notably increased the amount of specific radioligand binding in both mouse lines (p < 0.001). The increased binding was maximally 240 ± 23% of control binding without AA29504 in δKO mice, significantly higher than that in WT mice (166 ± 5% of control) (p < 0.001, Two-way ANOVA followed by Tukey’s post hoc test) (Fig. 6A).

Similar to the association, the dissociation rate constant K_off_ of [^3^H]muscimol binding at 22 °C was higher in δKO (0.66 ± 0.03 min^−1^) than in WT (0.38 ± 0.02 min^−1^) (mean ± SEM, n = 3), reflecting a faster radioligand dissociation in the former mouse line (p < 0.05, One-way ANOVA followed by Tukey's post hoc test). However, no evident effects were observed with AA29504 on [^3^H]muscimol.

K_off_ in δKO (0.64 ± 0.05 min^−1^) and WT forebrains (0.43 ± 0.02 min^−1^) (mean ± SEM, n = 3) (p > 0.05, One-way ANOVA followed by Tukey's post hoc test) (Fig. 6C).

### Saturation Analysis of [^3^H]muscimol Binding

**Fig. 7:**
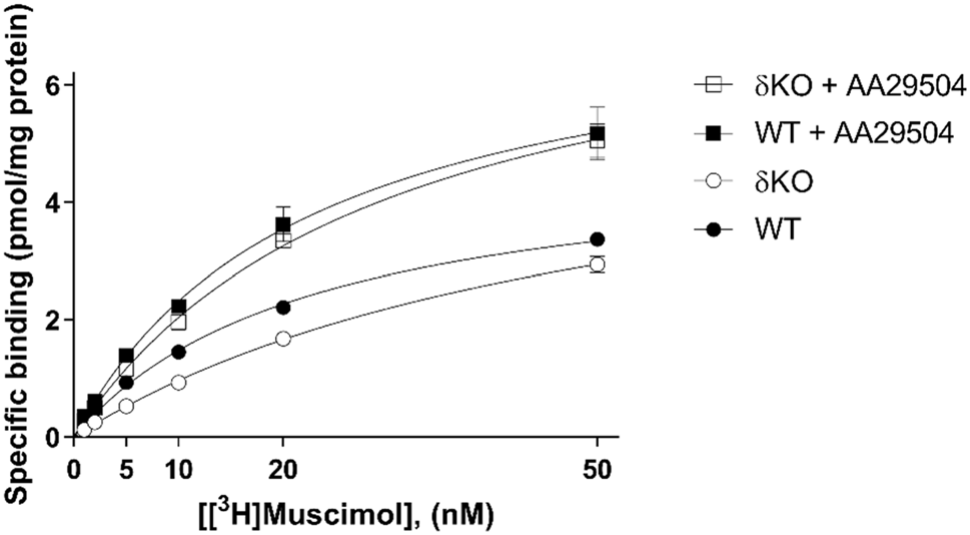
Saturation analysis of [^3^H]muscimol (1–50 nM) binding to WT and δKO mice forebrain membranes in the absence or presence of 10 µM AA29504. The values are expressed as pmol/mg protein (mean ± SEM, n = 3 independent experiments using triplicate membrane samples pooled individually from each mouse line’s forebrain). See Table 1. for detailed statistical comparisons and significance

As a probable mechanism for AA29504-induced stimulation of [^3^H]muscimol binding to GABA_A_ receptors, we hypothesized AA29504's potential to modulate agonist binding by shifting the binding affinity of specific receptor populations. Hence, we assessed the effects of AA29504 on the total number of high-affinity [^3^H]muscimol binding sites and binding affinity by analyzing the saturation kinetics of the high-affinity [^3^H]muscimol binding to WT and δKO mouse forebrain membranes at 22 °C (Fig. 7). Using increasing concentrations of [^3^H]muscimol (1–50 nM), in the absence (control) or presence of AA29504, all tested groups except control WT membranes were best fit to the one-site binding model. Binding to control WT membranes was best fit to the two-site binding model [p = 0.0013, F (DFn, DFd) = 11.11 (2, 14)], displaying two binding affinities at distinguishable receptor densities (Table 1). The sum of high affinity Bmax values (Bmax_(1)_ + Bmax_(2)_) in WT membranes, however, was equivalent to control δKO Bmax (no AA29504). The presence of 10 µM AA29504 in WT membranes rendered [^3^H]muscimol binding more favorable to one-site model as it displayed a single apparent affinity that was intermediate between the affinities obtained in control WT membranes. On the other hand, AA29504 significantly decreased the equilibrium dissociation constant (K_D_) reflecting an enhancement of [^3^H]muscimol binding affinity in δKOs (p < 0.001). Moreover, AA29504 increased [^3^H]muscimol Bmax in δKO (p < 0.05) as well as WT mouse lines (p < 0.01) (Two-way ANOVA followed by Tukey's post hoc test). The calculated [^3^H]muscimol Bmax and K_D_ values are summarized in Table 1 (Fig. 7).

**Table 1:** Values of equilibrium dissociation constants (K_D_) and total amount of high-affinity binding sites (B_max_), calculated from the saturation analysis of [^3^H]muscimol binding to WT and δKO mouse forebrain membranes at room temperature (22 °C)

Mouse line	AA29504 (10 µM)	K_D_ (nM)	B_max_ (pmol/mg protein)
WT	−	4.1 ± 3.6 (1), 40 ± 15.5 (2)	0.7 ± 0.7 (1), 5 ± 0.3 (2)
	+	23 ± 4	7.6 ± 0.9**
δKO	−	53 ± 6	6.1 ± 0.6
	+	30 ± 3^***^	8.1 ± 0.6*

K_D_ and B_max_ values represent means ± SEM, n = 3 independent experiments using triplicate membrane samples pooled individually from each mouse line’s forebrain

*p < 0.05, **p < 0.01, ***p < 0.001, significantly different from the corresponding control values without AA29504 (Two-way ANOVA followed by Tukey's post hoc test)

## Discussion

This paper probes into the complex action of the retigabine synthetic analogue, AA29504, on GABA_A_R binding properties and function. Using radioligand binding assays, we demonstrate the positive allosteric modulation of AA29504 on GABA- and THIP- induced responses in native GABA_A_Rs expressed in C57BL/6 J mouse forebrains. These modulatory activities are evident in WT and δKO mice with no differences in terms of potency. The results are not surprising as the ion-channel-site and allosteric modulation of the GABA and ion-channel coupling are relatively little changed in δKO mice [43]. The non-differential AA29504 potency between native GABA_A_Rs expressed in WT and δKO mice corresponds with the earlier observed modulation in αβ and αβγ recombinant expression systems [24, 27], leading to the conclusion that AA29504 is not particularly selective to αβδ GABA_A_Rs. However, as demonstrated with [^3^H]EBOB binding to recombinant GABA_A_Rs in this study, and similar to the earlier findings with electrophysiological measurements [27], AA29504 agonist efficacy is higher in α6β3δ than in α6β3γ2 receptors as it was more efficient in displacing [^3^H]EBOB directly (in the absence of GABA) in the former subtype (Fig. 3). Receptor desensitization was found to be a key factor determining GABA_A_Rs response efficacy [44–46]. The fact that δ-GABA_A_Rs display a slow desensitization rate and high open-channel stability [8, 47, 48] may contribute to the higher efficacy of AA29504 in relation to γ2-GABA_A_Rs. AA29504 agonist behavior, nevertheless, was not dependent on the presence of γ2 and δ subunits and even displaced [^3^H]EBOB with higher efficiency in α6β3 compared to α6β3δ and α6β3γ2 receptors. This further suggests the role of GABA_A_R’s transmembrane β + /α − interface in exerting AA29504 pharmacological activity [27, 49], as similarly found for neurosteroids [50, 51] and general anesthetics such as etomidate and propofol [52, 53]. Functional assessment at numerous mutant GABA_A_Rs and on in silico analysis of its low-energy conformations indicated that AA29504 and etomidate exert their effects through the same site or overlapping binding sites between α-TM1 and β-TM3 transmembrane domains [27]. Propofol and potentiating neurosteroids also bind between the same domains but at distinct binding pockets [54]. This inter-transmembrane binding site is in the vicinity of the physical desensitization gate at the intracellular end of the GABA_A_R channel [55–57] suggesting that the site could act as a target for modulating desensitization by AA29504, a mechanism of action already established for desensitization-modifying allosteric modulators (DAM) such as etomidate [58, 59], propofol [60, 61] and neurosteroids [62, 63].

Modulating GABA_A_R desensitization involves alterations in ligand binding properties as receptor affinity depends on its channel physical state according to the following order: resting state < open state < desensitized state [64, 65]. Thus, we examined AA29504 modulation of agonist binding to GABA_A_Rs where AA29504 increased the high-affinity [^3^H]muscimol and [^3^H]GABA binding to native GABA_A_Rs expressed in WT and δKO mouse forebrains. Mammalian WT fore/midbrain contain up to 10% of δ-GABA_A_Rs [66, 67] and the deletion of δ subunit in δKO mice leads to an increase in αβγ2 receptor expression since δ subunit does not compete with γ2 in receptor assembly with α and β subunits [68]. Despite the well-established high-affinity muscimol and GABA binding to δ-GABA_A_Rs [8, 10, 36, 41], the enhancements of GABA_A_R agonists binding were higher in δKO than in WT mice. The effect of AA29504 on [^3^H]muscimol binding was in fact absent in α6β2δ, while evident in α1β2γ2 and α6β2γ2 recombinant GABA_A_Rs (Fig. 5), suggesting the involvement of γ2-GABA_A_Rs in this enhancement. In binding kinetics assays, [^3^H]muscimol association and dissociation rates are exceptionally low in αβδ receptors, reflecting the slow binding and unbinding kinetics of muscimol in WT compared to δKO forebrain and cerebellar membranes (Fig. 6; [41]). This behavior was not altered upon co-incubation with AA29504 as we did not observe any significant changes in the association (K_on_) and dissociation (K_off_) rate constants in either mouse line. Hence, the link between AA29504-induced stimulation of [^3^H]muscimol binding and alterations in receptor-ligand binding kinetics was not established.

In agreement with AA29504 stimulation of the agonist binding, [^3^H]muscimol saturation analysis revealed AA29504-induced GABA_A_R shift to the high-affinity states. In WT mice, [^3^H]muscimol displayed two high-affinity receptor populations in the absence of AA29504: low-nanomolar (K_D_ = 4.1 ± 3.6 nM) for δ-GABA_A_Rs as earlier reported [41, 69], and intermediate-nanomolar (K_D_ = 40 ± 15.5 nM) for non-δ-GABA_A_Rs. The intermediate-nanomolar affinity in WT, comparable to δKO mice (K_D_ = 53 ± 6), was found to represent < 10% of total non‐δ‐GABA_A_Rs when occupied by 5 nM [^3^H]muscimol [41]. This non‐δ‐GABA_A_R population was suggested to rise from desensitized γ2-GABA_A_Rs [70], pre-frozen brain membranes at − 70 °C [71] and trace residual [GABA] from adequately washed brain membranes. Previous autoradiography and membrane homogenate binding assays showed that the deletion of δ subunit in δKO mice leads to a substantial loss of high‐affinity [^3^H]muscimol binding, especially in the forebrain region [12, 41, 43, 72], whereas in recombinant GABA_A_Rs, the replacement of γ2 subunit by δ in α6β2γ2 receptors abolished AA29504 enhancement in [^3^H]muscimol binding (Fig. 5). Therefore, the increase in [^3^H]muscimol Bmax in WT forebrain may be attributed to AA29504-induced alteration of non-δ-GABA_A_Rs towards a higher affinity state. This was confirmed upon co-incubation of AA29504 with δKO forebrain membranes which displayed an increase in the receptor sites available for high-affinity [^3^H]muscimol binding. These additional sites that were undetectable in the absence of AA29504 appeared as a result of an enhancement in [^3^H]muscimol binding affinity. It was reported earlier that δKO dentate gyrus granule cell macrocurrents exhibit considerably higher channel desensitization compared to WT [73]. Hence, a plausible explanation for this subtype-dependent [^3^H]muscimol binding is that αβδ receptors already exhibit high affinity to [^3^H]muscimol and undergo minor desensitization [41, 62, 74, 75] that is unaltered by AA29504. On the other hand, a part of low-affinity αβγ2 receptors with micromolar K_D_ desensitize upon AA29504 exposure and shift to a high-affinity state [70, 76] resulting in increased [^3^H]muscimol binding that can be measured in the nanomolar range (Fig. 4; [70]). These high-affinity [^3^H]muscimol-bound receptors display a desensitized non-functional state that is impermeable to chloride influx [69, 77]. However, this state is not permanent as ligand-bound desensitized receptors may re-sensitize and shift to a functional open state [78, 79]. This re-sensitization was found to increase the probability and mean time of GABA_A_R’s open state, which contributes to the prolongation of the inhibitory postsynaptic currents (IPSCs) [3, 80, 81]. Recent evidence has shown that desensitization promotes GABA_A_R phosphorylation by protein kinase C (PKC) leading to the rise of a new receptor population that induces long-term potentiation at the inhibitory synapses [82]. The phosphorylation by PKC was also reported to decrease GABA_A_R sensitivity to ethanol and benzodiazepines [83]. Hence, the influence of AA29504 on phosphorylation as a consequence of receptor desensitization needs to be examined for its potential role in regulating the allosteric modulatory effects on GABA_A_Rs.

## Conclusion

This study sheds light on AA29504's modulatory activity, its direct actions and interactions with agonists in GABA_A_R complex. Using [^3^H]EBOB radioligand as a unique probe for assessing drug enhancement of GABA_A_R function, we demonstrated for the first time the non-differential AA29504 modulatory potency on native GABA_A_Rs expressed in WT and δKO C57BL/6J mice. We further displayed AA29504’s GABA-independent activity on recombinant GABA_A_Rs expressed in HEK293 cells, indicating higher selective agonist efficacy on δ-GABA_A_Rs in relation to γ_2_-GABA_A_Rs. Interestingly, AA29504 showed a concentration-dependent stimulation of GABA_A_ agonist binding to γ2 GABA_A_Rs but not to δ-GABA_A_Rs. This newly revealed selective modulation by AA29504 is attributed to its ability to shift the low-affinity γ_2_-GABA_A_Rs towards a higher affinity desensitized state, thereby rising new sites capable of binding GABA_A_R agonists with low nanomolar affinity. Hence, the potential of AA29504 to act as a desensitization-modifying allosteric modulator (DAM) of γ2-GABA_A_Rs deserves further investigation for its promising influence on shaping efficacy, duration and plasticity of GABA_A_R synaptic responses [46, 62, 81, 82, 84, 85].
